# Seroprevalence of anti-SARS-CoV-2 antibodies among blood donors from December 2020 to June 2021 in Koutiala district, Mali

**DOI:** 10.1371/journal.pgph.0001316

**Published:** 2023-01-05

**Authors:** Fara Wagbo Temessadouno, Jean Gilbert Ndong, Etienne Gignoux, Yves Coppieters, Alhassane Ba, Youssouf Diam Sidibe, Aminata Daou, Nada Malou, Idrissa Compaore, Tidiani Fane, Erica Simons, Francisco Luquero, Clair Mills, Komla Mawunya Vuti, Marie Hortense Nkokolo massamba, Sonia Guiramand

**Affiliations:** 1 Médecins Sans Frontières, Dakar, Senegal; 2 Épicentre, Paris, France; 3 Médecins Sans Frontières, Bamako, Mali; 4 School of Public Health, Université Libre de Bruxelles (ULB), Brussels, Belgium; 5 Ministry of Health, Bamako, Republic of Mali; 6 Médecins Sans Frontières, Koutiala, Mali; 7 Médecins Sans Frontières, Paris, France; Georgetown University, UNITED STATES

## Abstract

Severe acute respiratory syndrome coronavirus 2 (SARS-CoV-2) is the virus associated with coronavirus disease (COVID-19). At the time of the study, little data on the level of exposure of the population in Koutiala district in Mali to SARS-CoV-2 was available. Although blood donors are not representative of the general population, a COVID-19 seroprevalence estimate in this population was intended to assess the extent of community transmission, serve as a health alert system, and help guide the public health response. We measured seroprevalence of anti-SARS-CoV-2 antibodies using NG-Biotech SARS-Cov-2 RDT and ECLIA test between January and June 2020. This is a cross-sectional study of volunteer blood donors aged 18 to 60 years, independent of any previous COVID-19 disease. A stratified analysis of seroprevalence by month of sample collection and a comparison of the results of the NG-Biotech SARS-Cov-2 RDT with those of the ECLIA test was performed. The overall prevalence of antibodies to SARS-Cov-2 virus assessed by the NG-Biotech SARS-Cov-2 RDT was 24.6% (95% CI 21.8–27.4) and by the ECLIA test was 70.2 (95% CI 64.9–75.5). Both estimates remained relatively stable over the study period. We observed SARS-CoV-2 exposure much higher than indicated by case-based surveillance. The national surveillance system, as it was, was not able to detect variations in incidence, and as such, we do not recommend it as an alert system. However, the discrepancy between the results of the rapid test and the ECLIA test shows that further research is required to assess the validity of these test before a more solid conclusion can be drawn it their use in surveillance.

## 1. Introduction

Severe acute respiratory syndrome coronavirus 2 (SARS-CoV-2) is associated with coronavirus disease (COVID-19). It was first detected in December 2019 in Wuhan, China, and the World Health Organization (WHO) declared a global pandemic on 11 March 2020. At the time of proposal of this study in July 2020, more than 12.2 million confirmed cases and 550,000 deaths had been reported worldwide, including in many low- and middle-income countries [1]; however, many key epidemiological and serological characteristics of COVID-19 were still unknown, including its transmissibility and severity.

In addition to clinical surveillance based on confirmed cases, several countries (e.g., France, Kenya, United States, Denmark, Scotland) had already conducted seroprevalence studies to estimate the extent of the disease at the community level and monitor its spread over time [2–9]. These studies found seroprevalence estimates much higher than the number of reported cases in most locations, which can be attributed in part to asymptomatic or paucisymptomatic cases and the different screening strategies deployed [10, 11]. Different designs were used, including seroprevalence surveys among blood donors. Rapid and requiring few resources, this approach provided a proxy of seroprevalence at the community level in locations with limited screening capacity or access to health care.

The government of the Republic of Mali declared a public health state of emergency due to COVID-19 on 25 March 2020 after the detection of the first case. In May 2020, the first COVID-19 positive case was recorded in the district of Koutiala where Médecins Sans Frontières conducts health activities in collaboration with the Ministry of Health. Between May 2020 and January 2021, 254 PCR samples were collected in Koutiala and a total of 32 positive patients reported.

At the time of the study, few data were available on the level of exposure of the population of Koutiala district to SARS-CoV-2. As diagnosis of infection was limited and underreporting of cases likely. Less than 15,000 cases had been reported in Mali at the time of the survey and 23 cases in Koutiala, corresponding to attack rates of less than 0.1% and 0.002%, respectively.

A seroprevalence study was proposed to help assess the magnitude of the epidemic and to guide the health response. In collaboration with the MOH, MSF managed the blood bank of the Reference Health Centre (CSRef) in Koutiala. In 2020, the Koutiala blood bank distributed 7,400 units of blood to the entire hospital. Although blood donors are not representative of the general population, the purpose of having an estimate of prevalence in this population was to assess the extent of community transmission, to serve as a health alert system, and to help guide the public health response. The availability of new diagnostic tests also allowed an opportunity to compare immunological detection methods, so was included as part of the overall study.

## 2. Methods

### 2.1. Study design, site, and sampling

This cross-sectional study used exhaustive sampling of blood donors presenting to the CSRef blood bank in Koutiala; all donors were from Koutiala health district. This district is in the north of the Sikasso Region, 135 km from Sikasso, the regional capital.

MSF provides clinical care in the district, including supporting the blood bank. The study was conducted between December 2020 and June 2021; seroprevalence was estimated monthly.

All persons between the ages of 18 and 60 years who had volunteered to donate blood, regardless of previous COVID-19 disease, and who met the blood donor selection criteria as established by MSF and the Ministry of Health (MOH) were included in the study after giving their consent. For instance, each blood donor volunteer was allowed to donate blood only once in 3 months.

The sample size was calculated using OpenEpi software assuming a seroprevalence of 5%, a type 1 risk of error of 5% (α), a precision of 2.5%, and 5% invalid or refusal to participate in the study; this required analysis of a minimum of 307 samples each month to obtain prevalence estimates with this precision.

### 2.2. Sample collection

A trained nurse from the blood bank collected blood samples for transfusion-transmitted infections (TTI) screening and COVID-19 serological testing from the derivation pouch of anonymized blood units. The pre-donation questionnaire, modified to include information on COVID-19 symptoms (fever, cough, breathlessness), accompanied each blood sample.

### 2.3. Tests used in the study

#### Rapid diagnostic test (RDT) for COVID-19

At the time of the study, several qualitative membrane immunoassays, or rapid diagnostic tests (RDTs) for the detection of specific anti-SARS-CoV 2 antibodies with varying performance were becoming available on the market. In collaboration with Hospital Bicètre (Paris), MSF evaluated the performance of 10 RDTs. The specificity two weeks after symptom onset of the 10 selected tests ranged from 75.5% to 98.4% [12].

Among the 10 evaluated tests, the NG-TEST/ IgG-IgM COVID-19 (NG Biotech, Guipry France) was selected for its good performance (sensitivity (93.7%); specificity (99.2%)) ease of use (clarity of instructions and ease of interpretation of results) and commercial availability in relation to the worldwide demand at the time [12].

### 2.4. Procedure for using the rapid serological test

The NG-TEST/ IgG-IgM COVID-19 test targeting the Nucleocapsid protein was performed according to the manufacturer’s instructions by placing 10 μl of serum into the sample port (marked S on the cassette) and then adding dilution buffer. The results were read after 15 minutes, according to the manufacturer’s recommendations.

### 2.5. Inclusion of participants with positive malaria RDT

While initially excluded, patients with a positive malaria RDT test were included in the study from March 2021 onwards, after having obtained the agreement of the National Ethics Committee for Health and Life Sciences (CNESS) of Mali as well as MSF’s Ethical Review Board (ERB).

### 2.6. RT-PCR test

For patients with a SARS-CoV-2 RDT result positive for IgM only, a RT-PCR test was done at Centre of Infectiology Charles Merieux (CICM) in Bamako starting in late March 2021as soon as the logistical possibilities of transporting the samples were made possible.

### 2.7. Electrochemiluminescence immunoassay (ECLIA)

Starting in March 2021, the Elecsys Anti-SARS-CoV-2 Test, an immunological test for the qualitative determination of anti-SARS-COV-2 antibodies, was additionally performed on serum at the Pa et Ka laboratory in Bamako.

The result is expressed as a cut-off index (CQI), a value calculated by dividing the electrochemiluminescence signal of the sample with the signal obtained by calibration, the cut-off is 1 CQI. As reported by the manufacturer, this test has a sensitivity of 99.5% (95% CI 97–100) and a specificity of 99.8% (95% CI 99.7–99.9) [13].

### 2.8. Data collection and management

According to routine procedures, each participant received a clinical examination and was administered a pre-donation questionnaire that had been modified for the study to include questions related to COVID-19. The interviews were conducted in Bambara. Each donor was informed about the TTI screening, including HIV, Hep B, Hep C, Syphilis, Malaria, and the additional COVID-19 serological test.

Data, including questionnaire responses and laboratory results, were collected using electronic forms (Kobo collect) on password-protected mobile devices (Samsung pad). Data were regularly uploaded to a password-controlled Epicentre server and routine data checks were performed.

Blood bank registers were used to capture data following standard laboratory procedures. The study forms were linked by a unique identification number assigned to each study participant.

### 2.9. Data analysis and statistical methods

Data were analysed using R version 4.1.1 (The R Foundation for Statistical Computing, Vienna Austria). Descriptive analysis, including age and sex, was calculated as proportions. For the estimation of seroprevalence, a positive RDT result was defined as positive IgM, positive IgG or positive IgM and IgG. The main outcome of interest is the estimate of seroprevalence over the study period. An additional stratified analysis of seroprevalence by month of sample collection was performed. Two-sample Student’s t-test and Pearson’s chi-square statistics were used to compare continuous and categorical descriptive results, respectively. We explored the relationship between the RDT result, age, gender and the ECLIA signal value in univariate analysis considering the 20% threshold. Logistic regression analysis was performed to analyse the relationship between the ECLIA test value signal and the NG TEST result. Results were calculated with 95% confidence intervals.

### 2.10. Ethical considerations

The study protocol was approved by the MSF Ethics Review Board (ERB) and by Mali’s National Ethics Committee for Health and Life Sciences (CNESS). The participants were voluntary adult donors who provided written informed consent.

The study was conducted in accordance with the international ethical guidelines of the Council for International Organizations of Medical Sciences (CIOMS) for biomedical research involving human subjects, the international ethical guidelines for epidemiological studies, and Decree N°0245/P-RM of 13 March 2017 setting out the terms of application of Law N°09–059 of December 2009 governing biomedical research on human beings in Mali.

## 3. Results

### 3.1. Study flow chart

**Fig 1:**
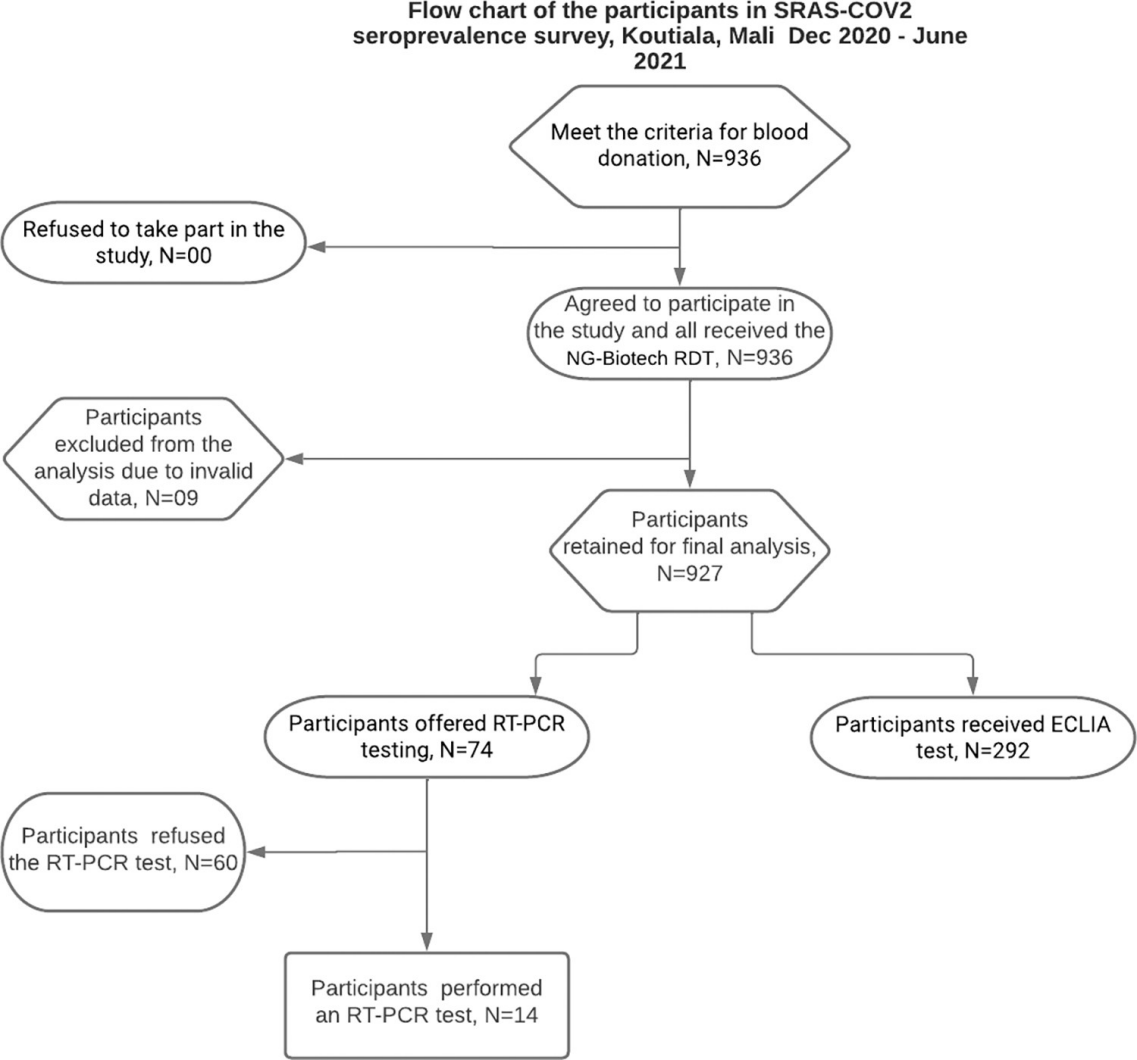
Seroprevalence of anti-sars-cov-2 antibodies among blood donors participants’ flow chart.

A total of 936 people were enrolled in the study from December 2020 to June 2021, of whom 9 were subsequently excluded from the analysis due to poor data quality. No blood donor refused participation in the study. The analysis included 927 participants (Fig 1).

### 3.2. Participants’ characteristics

The average age of the participants was 32 years and 97% of whom were male (Table 1).

**Table 1:** Participants’ characteristics.

Participants’ Characteristics	N = 927
Age, (Median age, [IQR])	30 [25–38]
Male, n (%)	895 (96.5)
Previous blood donation	
*Never donated blood*, *n (%)*	*540 (58*.*0)*
*Already donated blood more than three months ago*, *n (%)*	*387 (42*.*0)*
Weight, (Median weight, [IQR])	69 [63–76]
*Haemoglobin level (Median haemoglobin, [IQR])	14.7[13.9–15.6]

*Data on haemoglobin was inconsistent for 14 participants

### 3.3. Overall and monthly seroprevalence and prevalence according to the NG-Biotech RDT and ECLIA tests (Elecsys Anti-SARS-CoV-2 test)

The overall prevalence of antibodies to SARS-CoV-2 virus assessed by RDT was 24.6% (Table 1). Excluding those who were IgM positive only, the seroprevalence excluding these donors and the seroprevalence was 18.1% [95% CI 15.4–20.6]

The prevalence of antibodies assessed by the ECLIA test, available from March to June, is 70.2% and varies little by month (Table 2).

**Table 2:** Overall and monthly seroprevalence by type of test (RDT and ECLIA).

Month	Number of rapid antibodies tests	IgM +	IgG	IgM+ and IgG+	Total	RDT seroprevalence % [95% CI]	ECLIA: seroprevalence % [95% CI])	
Dec 20	76	11	2	10	**23**	30.3 [19.9–40.6]	NA	NA
Jan 21	132	7	6	21	**34**	25.7 [18.1–33.4]	NA	NA
Feb 21	250	27	10	24	**61**	24.4 [19.1–29.7]	NA	NA
March 21	222	15	4	28	**47**	21.2 [15.8–26.5]	64	68.5 [57.4–80.1]
April 21	90	10	1	13	**24**	26.7 [17.4–35.9]	84	64.2 [53.9–74.6]
May 21	88	1	1	20	**22**	25.0 [15.9–34.1]	87	75.7 [66.8–84.9]
June 21	69	3	3	11	17	24.6 [14.4–34.9]	57	71.9 [60.2–83.7]
**Total**	**927**	**74**	**27**	**127**	**228**	**24.6 [21.8–27.4]**	**292**	**70.2 [64.9–75.5]**

Of 292 participants who received both tests, 205 were seropositive by ECLIA t; of these, only 68 were positive by RDT. Of 87 participants who tested negative by ECLIA, only 7 were positive by the RDT i.e. Cohen’s Kappa concordance coefficient is 0.18 [0.11–0.24], p<0.003.

Among 74 participants who presented a positive RDT only for IgM, 14 carried out a PCR test, among which 4 (28.6%) had a positive PCR test.

### 3.4. Result of the NG TEST / IgG-IgM COVID-19 RDT according to the I/O signal in ECLIA test

Of 144 participants who were ECLIA positive with an ECLIA signal value available, 58% were positive by RDT. This varied from 13% when the ECLIA signal value was between zero and 10 to 63% when the ECLIA signal value was between 50 and 300 (Table 3).

**Table 3:** Result of the RDT according to the I/O signal in ECLIA test.

Signal value in ECLIA	N	RDT positive, n (%)	OR	P-value
0 - <10	47	6 (13)	ref	ref
10 - <20	30	7 (23)	2.1	0.2333
20 - <50	26	10 (38)	4.3	**0.0146**
50 - < 300	41	26 (63)	11.8	**<0.0005**
Total	144	84(58)		

### 3.5. Longitudinal monitoring of seroprevalence in relation to the national surveillance system

**Fig 2:**
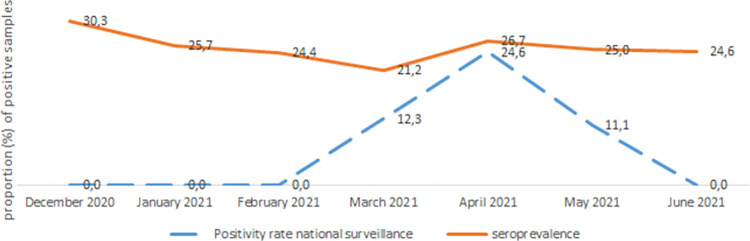
Comparative evolution of prevalence seroprevalence survey versus national surveillance systems, Koutiala, Mali, December 2020 to June 2021. Longitudinal monitoring at national level of the proportion of positive COVID-19 PCR tests, Mali national surveillance system and seroprevalence survey results for the same period.

Seroprevalence was highest in December 2020, declined slightly until March 2021 before increasing in April and levelling out between April and June 2021. According to the national surveillance system, no COVID-19 cases were detected in Koutiala until March 2021 before a slight peak in April (Fig 2). During the study period, of 146 COVID-19 PCR tests done in Koutiala health district, 23 of them (15.8%) were positive.

### 3.6. Rapid diagnostic test for malaria versus COVID-19 RDT

No significant difference was observed in COVID seroprevalence (RDT) according to malaria RDT result (28% among malaria RDT positive participants vs 25% among malaria RDT negative participants) (Table 4).

**Table 4:** Malaria RDT results versus COVID-19 RDT result.

Malaria RDT (HRP2 SD BIOLINE)	N	NG TEST COVID-19 positive, n (%)	P-value
Positive malaria RDT	25	7(28)	0.81
Negative malaria RDT	306	77(25)	
Total	331	84(25)	

## 4. Discussion

### 4.1. Overall seroprevalence

Our study found a high seroprevalence of anti-SARS-CoV-2 antibodies in Koutiala blood donors: 24.6% by RDT and ECLIA results almost 3 times higher (70.2%) between March and June 2021, a trend that was stable during the study period.

The availability of ECLIA testing in Bamako led us to introduce an ECLIA test for all participants after amending the protocol in this sense and validation by the various ethics committees. This explains the lower number of ECLIA tests compared to the NG-BioTech RDT.

### 4.2. Comparison of seroprevalence and reported cases / national surveillance system

There is a very low attack rate of 0.002% in Koutiala over the period according to the national surveillance system while the seroprevalence from survey remains high. This difference can be explained by the fact that the former detects ongoing symptomatic infection while the latter detects old and current cases. In addition, plausible differences between the blood donor population and the general population, lack of testing and asymptomatic cases might have contributed to widen this difference. The lack of specificity of the test in this population due to cross reactivity to other infection cannot be ruled out [14]. Havers FP, *et al* observed a difference between the prevalence observed in blood donors and the number of cases reported in the population [2], but not of this magnitude. This suggests that the difference is largely due to under-detection of cases by the surveillance system. On the other hand, the seroprevalence estimated from this study is prone to possible bias as blood donors were mostly men (97%). Though this study was not designed to investigate reason for this demography, this appears to be related to cultural specificities as well as certain health-related issues for women, including haemoglobin levels, pregnancy, and lactation.

### 4.3. Different testing methods

Several studies in developing countries have shown the limitations of RDTs and concluded it is not the preferred tool for active surveillance [15, 16]. Other studies evaluating the elycsis ELICSYS have shown good levels of performance and reliability [17, 18]. The performance of RDT has not been evaluated several months after the infection. The introduction of the ECLIA test during the study allowed us to increase the sensitivity and shown a low concordance between the 2 tests. Test performance may vary with time since infection and severity of infection, and ECLIA and RDT performance may relate differently to these aspects.

We observed that, although the ECLIA is described as a qualitative test, the higher the signal density of the test result, the higher the capacity of the RDT to detect anti-SARS-CoV-2 antibodies. The capacity of the NG-Biotech RDT to detect anti-SARS-CoV-2 antibodies was approximately 12 times higher if the ECLIA test signal value was greater than or equal to 50 compared to if it was less than or equal to 10.

At the same time as the ECLIA test was introduced, a PCR test was introduced under the same conditions and for the same reason. It was offered to all participants detected as IgM positive with the NG-Biotech RDT. Only a very small number of the participants concerned accepted to do the PCR test. Indeed, the national policy at that time would have been that anyone with a positive PCR test should be isolated in a treatment centre and our participants did not want to be isolated, especially as they were all asymptomatic. This would explain the fact that we do not have enough PCR to draw any conclusions.

### 4.4. Relationship between the rapid malaria diagnostic test, NG-Biotech SARS-CoV-2 RDT and the Elecsys Anti-SARS-CoV-2 test

One article reported cross-reactivity between IgM detected and sera with positive malaria serology [2]. However, our analysis did not find any relationship between positive malaria RDT test and NG-Biotech SARS-CoV-2 RDT or ECLIA Elecsys Anti-SARS- CoV-2 COVID-19 test, suggesting that there might not be cross reactivity between positive malaria test and NG-Biotech SARS-CoV-2 RDT results. This finding cannot be conclusive because of low statistical power due to very small sample size.

### 4.5. Validity and possible limitations

Antibody titres appear to be lower in asymptomatic and mild COVID-19 patients, the performance of these tests in this population and the decrease in antibody titre over time is a major limitation for accurate interpretation of serological results when used several weeks after exposure and in non-severe patients.

The expected number of study participants was not reached due to the exclusion of participants who had a positive rapid malaria test at the start of data collection. The smaller sample size has resulted in a widening of the confidence interval of our prevalence estimate, a precision of 2.8 instead of the expected 2.5. However, this does not change the main conclusions of our study which found much higher prevalence than expected based on the number of cases reported. While the sample size obtained was sufficient to obtain valid prevalence estimate, excluding these patients may have introduced a selection bias. However, no correlation observed between malaria status and seropositivity.

## 5. Conclusion

The prevalence of anti-SARS-CoV2 antibodies among blood donors in the Koutiala health district during the study period was high at 25% according to the NG-Biotech SARS-CoV-2 RDT and even higher at 70% according to the Elecsys Anti-SARS-CoV-2 Test, which suggests widespread circulation of the virus in in Koutiala and remained stable during the study period. National surveillance system was unable to detect variations in incidence and as such we do not recommend it as an alert system. However, the discrepancy between the results of the rapid test and the ECLIA test shows that further research is required to assess the validity of these test before a more solid conclusion can be drawn it their use in surveillance.

## References

[1] Johns Hopkins Coronavirus Resource Center. COVID-19 Map [Internet]. Available from: https://coronavirus.jhu.edu/map.html

[2] Havers FP, Reed C, Lim T, Montgomery JM, Klena JD, Hall AJ, et al. Seroprevalence of Antibodies to SARS-CoV-2 in 10 Sites in the United States, March 23-May 12, 2020. JAMA Intern Med. 2020 Dec 1;180(12):1576. doi: 10.1001/jamainternmed.2020.4130 32692365 PMC12507447

[3] Slot E, Hogema BM, Reusken CBEM, Reimerink JH, Molier M, Karregat JHM, et al. Herd immunity is not a realistic exit strategy during a COVID-19 outbreak [Internet]. In Review; 2020 Apr [cited 2022 May 25]. Available from: https://www.researchsquare.com/article/rs-25862/v1

[4] Valenti L, Bergna A, Pelusi S, Facciotti F, Lai A, Tarkowski M, et al. SARS-CoV-2 seroprevalence trends in healthy blood donors during the COVID-19 outbreak in Milan. Blood Transfus [Internet]. 2021 Apr 28 [cited 2022 May 25]; Available from: doi: 10.2450/2021.0324-20 33539289 PMC8092034

[5] Thompson C. et al. Neutralising antibodies to SARS coronavirus 2 in Scottish blood donors—a pilot study of the value of serology to determine population exposure. [Internet]. 2020. Available from: https://pesquisa.bvsalud.org/portal/resource/pt/ppmedrxiv-20060467?lang=en

[6] Erikstrup C, Hother CE, Pedersen OBV, Mølbak K, Skov RL, Holm DK, et al. Estimation of SARS-CoV-2 Infection Fatality Rate by Real-time Antibody Screening of Blood Donors. Clin Infect Dis. 2021 Jan 27;72(2):249–53. doi: 10.1093/cid/ciaa849 33501969 PMC7337681

[7] Sood N, Simon P, Ebner P, Eichner D, Reynolds J, Bendavid E, et al. Seroprevalence of SARS-CoV-2–Specific Antibodies Among Adults in Los Angeles County, California, on April 10–11, 2020. JAMA. 2020 Jun 16;323(23):2425. doi: 10.1001/jama.2020.8279 32421144 PMC7235907

[8] Grzelak L, Temmam S, Planchais C, Demeret C, Tondeur L, Huon C, et al. A comparison of four serological assays for detecting anti–SARS-CoV-2 antibodies in human serum samples from different populations. Sci Transl Med. 2020 Sep 2;12(559):eabc3103. doi: 10.1126/scitranslmed.abc3103 32817357 PMC7665313

[9] Uyoga S, Adetifa IMO, Karanja HK, Nyagwange J, Tuju J, Wanjiku P, et al. Seroprevalence of anti–SARS-CoV-2 IgG antibodies in Kenyan blood donors. Science. 2021 Jan;371(6524):79–82. doi: 10.1126/science.abe1916 33177105 PMC7877494

[10] Kevadiya BD, Machhi J, Herskovitz J, Oleynikov MD, Blomberg WR, Bajwa N, et al. Diagnostics for SARS-CoV-2 infections. Nat Mater. 2021 May;20(5):593–605. doi: 10.1038/s41563-020-00906-z 33589798 PMC8264308

[11] Li R, Pei S, Chen B, Song Y, Zhang T, Yang W, et al. Substantial undocumented infection facilitates the rapid dissemination of novel coronavirus (SARS-CoV-2). Science. 2020 May;368(6490):489–93. doi: 10.1126/science.abb3221 32179701 PMC7164387

[12] Dortet L, Ronat JB, Vauloup-Fellous C, Langendorf C, Mendels DA, Emeraud C, et al. Evaluating 10 Commercially Available SARS-CoV-2 Rapid Serological Tests by Use of the STARD (Standards for Reporting of Diagnostic Accuracy Studies) Method. Hanson KE, editor. J Clin Microbiol. 2021 Jan 21;59(2):e02342–20. doi: 10.1128/JCM.02342-20 33239381 PMC8111137

[13] Muench P, Jochum S, Wenderoth V, Ofenloch-Haehnle B, Hombach M, Strobl M, et al. Development and Validation of the Elecsys Anti-SARS-CoV-2 Immunoassay as a Highly Specific Tool for Determining Past Exposure to SARS-CoV-2. Loeffelholz MJ, editor. J Clin Microbiol. 2020 Sep 22;58(10):e01694–20. doi: 10.1128/JCM.01694-20 32747400 PMC7512151

[14] Bates TA, Weinstein JB, Farley S, Leier HC, Messer WB, Tafesse FG. Cross-reactivity of SARS-CoV structural protein antibodies against SARS-CoV-2. Cell Rep. 2021 Feb;34(7):108737. doi: 10.1016/j.celrep.2021.108737 33545052 PMC7835103

[15] Eyre DW, Lumley SF, O’Donnell D, Stoesser NE, Matthews PC, Howarth A, et al. Stringent thresholds in SARS-CoV-2 IgG assays lead to under-detection of mild infections. BMC Infect Dis. 2021 Dec;21(1):187. doi: 10.1186/s12879-021-05878-2 33602152 PMC7889711

[16] Pallett SJC, Rayment M, Patel A, Fitzgerald-Smith SAM, Denny SJ, Charani E, et al. Point-of-care serological assays for delayed SARS-CoV-2 case identification among health-care workers in the UK: a prospective multicentre cohort study. Lancet Respir Med. 2020 Sep;8(9):885–94. doi: 10.1016/S2213-2600(20)30315-5 32717210 PMC7380925

[17] Afzal N, Tariq N, Raza S, Shakeel D. Diagnostic Accuracy of Electro-Chemiluminescence Immunoassay Anti-SARS-CoV-2 Serological Test. Cureus [Internet]. 2021 Jan 9 [cited 2022 May 25]; Available from: https://www.cureus.com/articles/47641-diagnostic-accuracy-of-electro-chemiluminescence-immunoassay-anti-sars-cov-2-serological-test doi: 10.7759/cureus.12588 33575149 PMC7870122

[18] Weber MC, Risch M, Thiel SL, Grossmann K, Nigg S, Wohlwend N, et al. Characteristics of Three Different Chemiluminescence Assays for Testing for SARS-CoV-2 Antibodies. Liu Z, editor. Dis Markers. 2021 Jan 6;2021:1–13. doi: 10.1155/2021/8810196 33532006 PMC7834819

